# Glomerular diseases in a Hispanic population: review of a regional renal biopsy database

**DOI:** 10.1590/S1516-31802009000300006

**Published:** 2009-10-06

**Authors:** Luis Fernando Arias, Jorge Henao, Rubén Darío Giraldo, Nelson Carvajal, Joaquin Rodelo, Mario Arbeláez

**Affiliations:** 1 MD, PhD. Associate professor, Department of Pathology; and Director of PRYT Group, School of Medicine, University of Antioquia, Medellín, Colombia.; 2 MD. Titular professor, Section of Nephrology, School of Medicine, University of Antioquia, Medellín, Colombia.; 3 MD. Pathologist, Department of Pathology; and co-investigator in PRYT Group, School of Medicine, University of Antioquia, Medellín, Colombia; 4 MD. Auxiliary professor, Section of Nephrology, School of Medicine, University of Antioquia, Medellín, Colombia.; 5 MD. Nephrologist, Section of Nephrology, School of Medicine, University of Antioquia, Medellín, Colombia.; 6 MD. Titular professor, Section of Nephrology, School of Medicine, University of Antioquia, Medellín, Colombia.

**Keywords:** Epidemiology, Glomerulonephritis, Hispanic americans, Database, Biopsy, Epidemiología, Glomerulonefritis, Hispanoamericanos, Base de datos, Biopsia

## Abstract

**CONTEXT AND OBJECTIVE:**

Epidemiological data provide useful information for clinical practice and investigations. This study aimed to determine glomerular disease frequencies in a region of Colombia and it represents the basis for future studies.

**DESIGN AND SETTING:**

Single-center retrospective analysis at the University of Antioquia, Colombia.

**METHODS:**

All native renal biopsies (July 1998 to December 2007) were reviewed, but only glomerular diseases were analyzed. The diagnosis of each case was based on histological, immunopathological and clinical features.

**RESULTS:**

A total of 1,040 biopsies were included. In 302 cases (29.0%), the patient’s age was ≤ 15 years. Primary glomerular diseases were diagnosed in 828 biopsies (79.6%) and secondary in 212 (20.4%). The most common primary diseases were focal and segmental glomerulosclerosis (FSGS) (34.8%), immunoglobulin A (IgA) nephropathy (IgAN) (11.8%), membranous glomerulonephritis (MGN) (10.6%), minimal change disease (MCD) (10.6%), crescentic glomerulonephritis (GN) (5.6%), and non-IgA mesangial proliferative GN (5.6%). Postinfectious GN represented 10.7% of the diagnoses if included as primary GN. Lupus nephritis corresponded to 17.8% of the entire series. In adults, the order of the most frequent primary diseases was: FSGS, IgAN, MGN, crescentic GN and MCD. In children (≤ 15 years), the most frequent were: FSGS, postinfectious GN, MCD, non-IgA mesangial proliferative GN, endocapillary diffuse GN and IgAN.

**CONCLUSIONS:**

As among Afro-Americans, FSGS is the most frequent type of glomerulopathy in our population, but in our group, there are more cases of IgAN. The reasons for these findings are unclear. This information is an important contribution towards understanding the prevalence of renal diseases in Latin America.

## RESUMEN

**CONTEXTO Y OBJETIVO:**

Los datos epidemiológicos dan información útil en clínica e investigación. Nuestro objetivo fue determinar frecuencias de enfermedad glomerular en una región de Colombia y representa la base para trabajos futuros.

**DISEÑO Y UBICACIÓN:**

Análisis retrospectivo en un único centro: Universidad de Antioquia, Colombia.

**MÉTODOS:**

Todas las biopsias de riñón nativo fueron revisadas (1998 - 2007), pero solo analizamos enfermedades glomerulares. El diagnóstico en cada caso estuvo basado en histología, inmunopatología y características clínicas.

**RESULTS:**

1.040 biopsias fueron incluidas. En 302 casos (29,0%) la edad del paciente fue ≤ 15 años. El diagnóstico fue enfermedad primaria en 828 biopsias (79,6%) y secundaria en 212 (20,4%). Las enfermedades primarias más frecuentes fueron glomeruloesclerosis focal y segmentaria (GEFyS) (34,8%), nefropatía IgA (NIgA) (11,8%), glomerulonefritis membranosa (GNM) (10,6%), enfermedad de cambios mínimos (ECM) (10,6%), glomerulonefritis (GN) extracapilar (5,6%) y GN proliferativa mesangial no-IgA (5,6%). La GN postinfecciosa representa el 10,7% de glomerulopatías primarias. La nefritis lúpica corresponde al 17,8% de todos los casos. En adultos el orden de frecuencia de enfermedades primarias es: GEFyS, NIgA, GNM, GN extracapilar y ECM. En niños (≤ 15 años) las más frecuentes fueron: GEFyS, GN postinfecciosa, ECM, GN mesangial no-IgA, GN endocapilar difusa y NIgA.

**CONCLUSIONES:**

Al igual que en afro-americanos, en nuestra población la GEFyS es la glomerulopatía más frecuente, pero en nuestro grupo hay más NIgA. Las razones para estos hallazgos no se conocen. La información presentada aquí es una contribución importante para el entendimiento de la prevalencia de enfermedades renales en Latinoamérica.

## INTRODUCTION

Studying the epidemiological aspects of renal diseases, both primary and secondary, may help to identify the frequency of glomerulopathy or other kidney diseases, their causes, the ethnic, environmental or genetic factors contributing towards disease development, the presenting symptoms, the potential regional difficulties, the local biopsy indications and other relevant clinical and histological features. Many reports dealing with national and regional databases, specific population groups, specific diagnoses or local single-center experiences have been published. A review of renal biopsy data may provide an insight into the spectrum of significant renal diseases within the community.

Our institution is a reference centre for the northwestern region of Colombia, in the province of Antioquia, which has a population of 5,672,000 inhabitants (2005). Here, we present the incidence of biopsy-proven renal diseases in our region, emphasizing the findings of primary and secondary glomerulopathies.

This study reports the frequencies of diagnoses among both children and adults, from renal biopsies in the pathology laboratory. Our pathology laboratory is the one that analyzes the greatest number of renal biopsies in the region and it is the only one where immunofluorescence (IF) is routinely performed.

## OBJECTIVE

Our aim was to study the epidemiology of renal diseases based on histological diagnoses, at a center with Hispanic patients in a region of Colombia.

## METHODS

This was a retrospective study from a single pathology laboratory. Renal biopsy specimens from children and adult patients with primary and secondary renal disease were included in the study. All the biopsies came from Hispanic patients living in our geographic region (northwestern Colombia) and were performed over a 9.5-year period (July 1998 to December 2007). They were evaluated by means of optical microscopy and IF, using standard procedures. Electron microscopy (EM) was available only at another center, for selected cases. The criteria for EM analysis were that no precise diagnosis had been obtained through optical microscopy and IF and that sufficient renal tissue was available for this ultrastructural study. If no final diagnosis was achieved through optical microscopy and IF, and EM could not be performed, the case was excluded from the final analysis.

A total of 1,185 native renal biopsies were examined over this 9.5-year period. All the biopsies were retrospectively reviewed by two pathologists (L.F.A. and R.D.G.) and were analyzed for this study. Diagnoses was made by consensus between the two pathologists. The renal biopsies were studied by means of routine staining: hematoxylin-eosin, Masson’s trichrome, periodic acid-Schiff and methenamine silver, and in selected cases, with other histochemical stains (Congo red and phosphotungstic acid-hematoxylin).

For cases without adequate frozen tissue for IF (i.e. frozen tissue without glomeruli or tissue fixed in formalin), the formalin-fixed, paraffin-embedded renal tissue was used for IF. Prior antigen retrieval with enzymatic digestion was performed as described by Nasr et al.^1^ However, instead of pronase, we use porcine trypsin, which is the enzyme available for antigen retrieval in our laboratory. This modification of the technique of Nasr et al. was proven and standardized in our laboratory.

The histological classification of renal diseases was based on the World Health Organization (WHO) recommendations.^2^ Clinical data were collected from the medical record archives. Primary or secondary diseases were diagnosed and classified based on clinical features, and laboratory tests were performed on all the material from these patients. The paraclinical evaluation included tests for hepatotropic viruses, HIV, serum complement levels, cryoglobulins, antinuclear antibodies (ANA) and other immunological tests. It also included serum protein electrophoresis and other tests according to the clinical features or biopsy diagnosis. The data on familial disease were incomplete, since no rigorous familial screening was done on most of our patients.

*Data analysis*. Descriptive statistics were used to present the distribution of the histological types of glomerulopathies and their relative frequencies. Age data were presented as means ± standard deviations (SD). The data were analyzed using the Statistical Package for the Social Sciences (SPSS®) software, version 11.5.

## RESULTS

Data were collected from 1,185 renal biopsies. Forty-eight cases (4.1%) had diagnoses of non-glomerular diseases: 33 cases of tubulointerstitial nephropathy (2.8%) and 15 cases of vascular nephropathy (1.3%). In 83 cases (7.0%), there was not enough tissue for a precise diagnosis (advanced sclerosing and unclassifiable lesions were included here), and in 14 cases (1.2%), no precise diagnosis could be achieved even though there was sufficient tissue material. Taking these 145 cases away from the 1,185 biopsies, a total of 1,040 biopsies were thus included in the present study. The analyses that follow were exclusively based on these 1,040 glomerulopathy cases.

**Figure 1.**
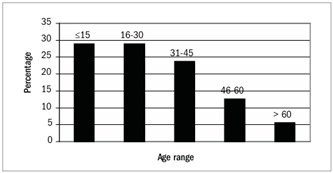
Distribution of age groups among patients with primary and secondary glomerulopathies (n = 1,040).

The patients’ mean age was 28.2 ± 17.6 years (range: 0-76; median 25.0). In 302 cases (29.0%), the age was ≤ 15 years, and in 58 cases (5.6%), the age was > 60 years. The distribution of renal biopsies according to the patients’ ages can be seen in Figure 1. 56.4% of the patients were female.

The most common indication for a biopsy was nephrotic syndrome, followed by non-nephrotic range proteinuria with or without hematuria, asymptomatic hematuria and acute renal failure.

The frequency of primary glomerular diseases was almost the same among females and males: 50.3% and 49.7% respectively. Secondary glomerular diseases were more frequent among females (71.8%). This figure rose to 81.1% if postinfectious glomerulonephritis (GN) was not included as a secondary glomerular disease.

The more frequent glomerulopathies (including both primary and secondary glomerular diseases) were focal and segmental glomerulosclerosis (FSGS) (288 cases; 27.7%), lupus nephritis (185 cases; 17.8%), immunoglobulin A (IgA) nephropathy (98 cases; 9.4%), postinfectious GN (89 cases; 8.6%), membranous GN (88 cases; 8.5%) and minimal change disease (88 cases; 8.5%). Considering only primary glomerulopathies (and including postinfectious GN among these), there was a total of 828 biopsies: FSGS accounted for 34.8%; IgA nephropathy, 11.8%; postinfectious GN, 10.7%; membranous GN, 10.6%; minimal change disease, 10.6%; crescentic GN, 5.6% (46 cases); non-IgA mesangial proliferative GN (not otherwise specified, NOS), 5.6% (46 cases); proliferative endocapillary GN (NOS), 5.0% (41 cases); and type I membranoproliferative GN, 4.1% (34 cases) (Table 1).

Among the patients with secondary glomerular disease, most had lupus nephritis: 185 cases, accounting for 17.8% of the entire series, 61.5% of secondary glomerulopathies if postinfectious GN was included as a secondary form and 87.2% if postinfectious GN was not included as a secondary form (Table 1).

Other less frequent glomerulopathies in our series, including both primary and secondary diseases, were: Henoch-Shönlein syndrome, 0.7%; thrombotic microangiopathy and hemolytic-uremic syndrome, 0.7%; amyloidosis, 0.6%; thin basement membrane disease, 0.6%; diabetic nephropathy, 0.4%; anti-glomerular basement disease, 0.2%; immunoglobulin (IgM) nephropathy, 0.2%; cryoglobulinemic GN, 0.1%; C1q nephropathy, 0.1%; and C3 nephropathy, 0.1%.

Table 1 presents the frequency analyses for children (≤ 15 years) and adults, separately.

**Table 1.** Frequency of primary and secondary glomerular diseases and their distribution among both adults and children

	Total n (%)	Adults: n = 738				Children: n = 302		
		% of primary or secondary cases	Subtotal n (%)	M n (%)	F n (%)	Subtotal n (%)	M n (%)	F n (%)
Primary glomerular diseases	828 (79.6)		n = 563	270 (48.0)	293 (52.0)	n = 265	142 (53.6)	123 (46.4)
FSGS	288 (27.7)	(34.8)	212 (37.7)	109	103	76 (28.7)	48	28
IgA nephropathy	98 (9.4)	11.8	88 (15.6)	43	45	10 (3.8)	3	7
Postinfectious GN	89 (8.6)	(10.7)	24 (4.3)	14	10	65 (24.5)	31	34
Membranous GN	88 (8.5)	(10.6)	79 (14.0)	35	44	9 (3.4)	3	6
Minimal change disease	88 (8.5)	(10.6)	33 (5.9)	18	15	55 (20.8)	32	23
Crescentic GN	46 (4.4)	(5.6)	38 (6.7)	12	26	8 (3.0)	4	4
Mesangial proliferative*	46 (4.4)	(5.6)	28 (5.0)	10	18	18 (6.8)	10	8
Endocapillary proliferative*	41 (3.9)	(5.0)	30 (5.3)	22	8	11 (4.2)	4	7
Membranoproliferative	34 (3.3)	(4.1)	25 (4.4)	7	18	9 (3.4)	5	4
Thin basement disease	6 (0.6)	(0.7)	4 (0.7)	0	4	2 (0.8)	1	1
Others	4 (0.4)	(0.5)	2 (0.4)	0	2	2 (0.8)	1	1
Secondary glomerular diseases	212 (20.4)		n = 175	34 (19.4)	141 (80.6)	n = 37	6 (16.2)	31 (83.8)
Lupus nephritis	185 (17.8)	(87.2)	159 (90.3)	22	137	26 (70.3)	1	25
Henoch-Schönlein purpura	7 (0.7)	(3.3)	1 (0.6)	0	1	6 (16.2)	1	5
Hemolytic-Uremic TMA	7 (0.7)	(3.3)	3 (1.7)	3	0	4 (10.8)	3	1
Amyloidosis	6 (0.6)	(2.8)	6 (3.4)	5	1	0 (0.0)	0	0
Diabetic nephropathy	4 (0.4)	(1.9)	4 (2.3)	3	1	0 (0.0)	0	0
Anti-GBM disease	2 (0.2)	(0.9)	1 (0.6)	0	1	1 (2.7)	1	0
Cryoglobulinemic GN	1 (0.1)	(0.5)	1 (0.6)	1	0	0 (0.0)	0	0

FSGS = focal and segmental glomerulosclerosis; GN = glomerulonephritis; TMA = thrombotic microangiopathy; M = male; F = female; IgA = immunoglobulin A; Anti-GBM = anti-glomerular basement membrane. *Not otherwise specified.

## DISCUSSION

This regional renal biopsy database has enabled us to ascertain the most frequent glomerulopathies in our region. All of the patients were people living in northwestern Colombia, and they represent a relatively homogeneous population. All of them were Hispanic, which constitutes an ethnic group, at least in our country (the information came from the medical chart). It consists of a particular mix of native (indigenous), African and Spanish and other Caucasian origins. The geographical origin of these patients allowed us to consider them to be Hispanic: physical appearance or skin color are poor predictors of genomic ancestry.^3^

At present, there is no national database of renal biopsies or glomerulopathies in Colombia, and we do not know of any other regional database in this country. Hence, this is the first reported renal biopsy database on our population, and we cannot say whether this sample represents the true frequency of glomerulopathies in this country.

FSGS is the commonest glomerulopathy among our population and represents 34.8% of primary glomerulopathies. This proportion is close to what has been reported for Afro-American patients.^4^^,^^5^ Other authors have reported similar frequencies of FSGS among Hispanics.^6^ However, differing from Afro-American patients, IgA nephropathy was not rare among our group of Hispanic patients, and it accounted for 15.6% of primary glomerulopathies among adults and 3.8% among children. Haas^7^ reported a 9.3% frequency of IgA nephropathy among Hispanics. In a large biopsy series, the frequency of IgA nephropathy among blacks was less than 1.4%.^7^^,^^8^ The reason for this high incidence of FSGS among our biopsied patients is unknown. It is possible that genetic or environmental factors, race or frequency of infections play a key role in this difference between populations.

Membranous GN and minimal change disease, which are two of the most prevalent histological diagnoses among nephrotic patients, accounted for nearly the same proportions of primary glomerulopathies as in series in Europe, United States, Australia and Asia,^9^^,^^10^^,^^11^^,^^12^^,^^13^^,^^14^^,^^15^ but membranous GN has higher proportions in the Italian database (23.4%)^16^ and the São Paulo database (20.7%).^17^ Membranoproliferative GN also accounted for similar proportions of primary GN, compared with series in Spain, Italy and Brazil.^14^^,^^16^^,^^17^ On the other hand, a much higher diagnosis rate for this condition was found in series from Romania (29.4%)^9^ and Lithuania (17.9%).^18^ The proportion of IgA nephropathy was lower than in series from Romania,^9^ Czech Republic,^10^ Australia,^11^ Denmark,^12^ United States,^13^ China,^14^ Italy,^16^ Brazil,^17^ France,^19^ Japan^20^ and Korea.^21^

The reason for including postinfectious GN in the primary glomerulopathy group in some of our analyses was that, in this disease, there are unknown factors causing the glomerular disease. Extrarenal manifestations (differing from the infection) are usually not detected. Hence, it is possible that, as in other primary glomerular diseases, glomerular factors or genetic predisposition are very important in developing this GN.

The ideal renal biopsy should be processed for analysis using optical microscopy, IF and electron microscopy. However, in many regions of Latin America, there are insufficient resources for ultrastructural renal biopsy examination. Therefore, our study was based predominantly on findings from optical microscopy and IF, analyzed by experienced renal pathologists. For some of our specimens, we stored renal tissue and used it for electron microscopy when we were unable to establish a diagnosis by means of optical microscopy and IF. In some other cases, electron microscopy was carried out on the paraffin-embedded renal tissue, thanks to collaboration with other centers in developed countries. Even so, we are aware that some diseases may have been underdiagnosed in our series, such as thin membrane glomerular disease.

The incidence of glomerular diseases varies according to the biopsy resources and biopsy policies. These are reflected in the histological diagnoses that are made. There is no universally valid “epidemiology” of glomerular disease.^22^ Some centers only take biopsies when the pathological diagnosis would affect the therapy, or in subjects with signs of progressive renal disease.^23^^,^^24^ Many differences in specific proportions (or incidence) of glomerulopathies can probably be explained by these confounding factors. In our center, renal biopsy is carried out on patients with any sign of renal dysfunction or proteinuria of any level. Nonetheless, among patients with hematuria alone, many nephrologists do not undertake renal biopsy. This may be a reason for the low incidence in our database of thin basement glomerular disease. The absence of cases of viral hepatitis-associated GN in our series could be because in patients with glomerular disease and the hepatitis B or C virus, a renal biopsy is not always carried out.

Thus, the different results in many reports worldwide could indicate bias in selecting patients for biopsy or resources for renal tissue study, or in other factors. Nevertheless, many differences are probably due to differences in population genetics, race, environmental factors, frequency of infection or biopsy rate. Therefore, our results are not relevant to other populations.

## CONCLUSION

In conclusion, FSGS is the commonest glomerulopathy diagnosed by means of biopsy, in both adult and child patients from our region. In decreasing order of frequency, the primary glomerulopathies found among adults are IgA nephropathy, membranous GN and crescentic GN; and among children, postinfectious GN, MCD and non-IgA mesangial proliferative GN. This study provides a contribution towards understanding the epidemiology of glomerular diseases in Latin America, with possible implications for the planning of future research.
